# Supplementary material for: Multigene panel next generation sequencing in metastatic colorectal cancer in an Australian population
Source: PLoS One. 2023 Oct 5;18(10):e0292087. doi: 10.1371/journal.pone.0292087 (PMC10553362; doi:10.1371/journal.pone.0292087)
Supplement: Attachment — Submitted filename: Response to Reviewers.docx [file pone.0292087.s002.docx]

# Response to Reviewers

**Comment 1:**

The authors present a mutational landscape analysis of metastatic CRC patients using a targeted sequencing panel. The results suggest the importance of RAS /RAS-P53 mutations on survival in stage IV patients. More importantly, this analysis shows the value of NGS sequencing panels for stratifying patients into clinically actionable sub-groups.

**Answer 1:**

We thank the reviewer for this positive feedback. There is a paucity of data in the Australian context on NGS testing in the colorectal space and thus our manuscript is novel in adding to that information. We feel that our cohort has a significant degree of multi-cultural variability, reflective of the modern Australian cohort, and thus is a relevant literature manuscript

Comment 2:

The work focuses on the importance of mutational profiling of patients to determine clinically actionable groups. It uses the survival dependence of KRAS-P53 mutations in an ethnically diverse cohort to show the utility of targeted NGS panels. It is important to expose this work and present the analytical strategy for other researchers/clinicians to consider. This multi-ethnic cohort represents the addition of new/novel data to the field.

Answer 2:

Again, we thank the reviewer for this positive feedback

Comment 3:

The data/analysis presented does support the conclusions from the paper. The sample sizes could be larger, but this is frequently observed with human subject data. Generally, the manuscript lacks some key and relevant references, and discussion of the trends in context of the current similar findings in the field. For example, the KRAS-P53 effect on survival is documented in CRC and other cancers. The analysis results could be probed in more detail and it is not stated whether this is the only mutation pattern that bore any effect on survival trends.

Answer 3:

Again, we thank the reviewer for this positive feedback. This cohort of 180 patients is one of the largest NGS testing samples of CRC done in Australia. We did not identify any other mutation patterns that affected survival. Of note, PIC3KA correlated with no difference in clinical outcomes and this has been mentioned in our manuscript.

Comment 4:

The sample size is small for statistical validity using Kaplain Meier statistics. For example, 14 patients with both RAS-P53 mutations in Fig 2a. However, CI values are given which helps support the analysis trend. This trend is not novel and has been observed in multiple other studies/spontaneous CRC subtypes. This should be discussed in the manuscript.

See:
<https://journals.lww.com/amjclinicaloncology/fulltext/2004/02000/simultaneous_mutations_in_k_ras_and_tp53_are.8.aspx> and <https://pubmed.ncbi.nlm.nih.gov/33351476/>

Answer 3:

Again, we thank the reviewer for this positive feedback. The reference “<https://pubmed.ncbi.nlm.nih.gov/33351476/>” mentioned is already cited in our discussion and we have made reference to previous manuscripts highlighint the poor prognostic trnd in RAS and P53 mutated CRC.

We did not include “[https://journals.lww.com/amjclinicaloncology/fulltext/2004/02000/ simultaneous_mutations_in_k_ras_and_tp53_are.8.aspx](https://journals.lww.com/amjclinicaloncology/fulltext/2004/02000/%20simultaneous_mutations_in_k_ras_and_tp53_are.8.aspx)” as the results are from 2004 and treatment outcomes from that period are dated and hard to correlate with current standard of care regimens.

Comment 4:

Were these the only patterns identified that correlated with survival from the current study/oncomine profiles ?

Answer 4:

We did not identify any other mutation patterns that affected survival. Of note, PIC3KA correlated with no difference in clinical outcomes and this has been mentioned in our manuscript.

Comment 5:

What was the breakdown of sex/ethnic groupings in the KRAS-P53 mutated sets ? vs random sampling.

Answer 5:

There was no grouping or random sampling applied in the cohort. We used 180 sequential samples of CRC.

Comment 6:

The manuscript was well written and easy to understand. The study goals were well described and the impact of the work clearly put into context with the current field.

Answer 6:

Again, we thank the reviewer for this positive feedback

Comment 7:

The research involves human subjects and consequently is exempt from the PLoS rules around making all datasets publicly accessible. The authors suggest that these data could be available on request. It is unclear why the data from this study might not be deposited in a protected-data site like dbGap or BioProject archive.

Answer 7:

We have uploaded a de-identified minimum data set as part of the supplemental material

Comment 8:

APC is the most frequently mutated gene in colon cancer, but it is not included in the panel. Do the authors have any information about APC mutational status of these samples? If yes, taking into account the APC mutations, whether the conclusion is different from the current one?

Answer 8:

APC genes are not mutated in colon cancer but are germline mutations that predispose to increased risk of colon cancer. NGS testing is done on somatic tumour tissue to look for tumour specific rather than germline specific mutations. Thus this comment is not relevant to our project with assesses the role of NGS testing in somatic tissue mutation identification in CRC.

Comment 9:

Is there any correlation of mutation loads/amount of mutated genes detected with the MMR status in the samples tested?

Answer 9:

No specific correlation is noted between tumour mutational burden and MMR – although this is not a key focus or specific to NGS testing and thus outside the scope of our paper

Comment 10:

Whether there are any correlation of the different mutations with the responses to any therapies the patients received?

Answer 10:

Upfront response rate to first line chemotherapy was not different between mutations but given the retrospective nature of the project this is a difficult question to answer. Prospective cohort studies are required to accurately assess tumour response using RECIST1.1 criteria to assess this and cannot be inferenced from our data.

Comment 11:

What kinds of mutations were detected by this method? Do these mutations include non-sense mutations or only include the ones that have been reported to alter the function of the genes? If non-sense mutations were included, the authors should consider separate them from the other damaging mutations.

Answer 11:

We have described the types and nature of mutation testing in our manuscript as follows: -“Next generation sequencing was performed with the Ion Torrent Genexus integrated next generation sequencer using the Oncomine Precision Assay^TM^, which is 50-gene NGS panel that was introduced to our laboratory in June 2021. Only DNA hotspot mutations and copy number variations (CNVs) were assessed. RNA sequencing for fusions was not performed. The Ion Torrent Genexus System fully automates the specimen-to-report workflow. It automates all steps of the targeted NGS workflow starting from extraction, purification and quantification of nucleic acid. The Genexus Purification System replaces sample prep by extracting and quantifying nucleic acids. This is followed by automation of the library preparation (including DNA synthesis), template preparation and sequencing. Lastly, primary data analysis is also automated and variant reporting for DNA applications are performed using the Oncomine^TM^ Reporter software, a Thermo Fisher supplied bioinformatics analytic tools. The reported results from the 50-gene Oncomine^TM^ Precision Assay are filtered to provide a summary of the variants and copy number variation as well as the variant allele frequency.”

Comment 12:

Related to the Introduction section, as far as I know, BRAF is a S/T kinase, and therefore the encorafenib is not a tyrosine kinase inhibitor.

Answer 12:

Amended; Encorafenib acts as an ATP-competitive RAF kinase inhibitor – we have updated the manuscript to reflect this.

Comment 13:

It should be CDKN2A instead of CDK2NA in Results 3.1.1.

Answer 13:

Amended; Thank you.

Comment 14:

Whether the difference of RAS mutations among diverse ethnic groups is statistically significant?

Answer 14:

There was a slightly higher proportion of RAS mutations in patients born in Australia compared with overseas but the results were not statistically significant

Comment 15:

PLOS authors have the option to publish the peer review history of their article ([what does this mean?](https://journals.plos.org/plosone/s/editorial-and-peer-review-process#loc-peer-review-history)). If published, this will include your full peer review and any attached files.

Answer 15:

Yes, happy for publication of peer review history
