# Supplementary material for: Multigene panel next generation sequencing in metastatic colorectal cancer in an Australian population
Source: PLoS One. 2023 Oct 5;18(10):e0292087. doi: 10.1371/journal.pone.0292087 (PMC10553362; doi:10.1371/journal.pone.0292087)
Supplement: Attachment — Submitted filename: Response to Reviewers v2.docx [file pone.0292087.s003.docx]

# Response to Reviewers

Comment 1:

The authors have addressed the outstanding queries in their responses, however, I'm not sure the minimal data added as supplementary materials is sufficient to support the findings in the manuscript. Were there any sample qc results for the patients samples ie. Depth and read numbers or similar?

Answer 1:

Depth and read numbers were not available on next generation sequencing reports. For example, I have attached a redacted copy of one example of the output next generation sequencing obtained. As you can see below, such information is not present.

Comment 2:

The only other query I have is that the confidence intervals appear to be shown only for one of the groups on the test... could the authors clarify if the baseline kras data a single sample or the CI interval not shown ?

Answer 2:

Apologies, that is an omission on our end. The confidence intervals are now included in the main text and figure.

Comment 3:

Following clarity in this aspect I would recommend the work be published.

Answer 3:

We thank the authors for their feedback and hope PLOS ONE agrees for publication.

Comment 4: In this recently revised manuscript, the authors have made significant strides in addressing the concerns I had previously raised. However, there remains an issue pertaining to APC mutations that I strongly disagree with the authors on. In their rebuttal letter, the authors assert that APC is exclusively subject to germline mutations and not somatic mutations in the context of colorectal cancer (CRC). I find this assertion problematic due to the well-established knowledge spanning over two decades, which affirms that somatic APC mutations are indeed among the earliest events in the development of non-hereditary colorectal carcinogenesis. These somatic mutations are pivotal in driving the formation of early adenoma/dysplastic crypt structures. While it is reasonable to acknowledge that the commercial 50-gene panel used in the Oncomine Precision AssayTM might not have initially incorporated APC, I believe that as professionals and investigators focused on CRC, the authors should have at least initiated a scientific discussion concerning the potential limitations of omitting this widely recognized driver gene from their study. It seems unlikely that the authors were unaware of prior genetic research pertaining to APC's role in CRC. With the exception of this concern, I do not have any further queries or reservations regarding the content of this manuscript.

Answer 4:

We thank the authors for their feedback. We agree with the reviewer that somatic APC mutations are among the earliest in the development of colorectal cancer. APC mutations are typically screened using specific PCR or NGS in certain subgroups.

Based on Australian guidelines, testing for APC using NGS is only indicated in patients who have isolated colorectal cancer under the age of 40, those with KRAS G12C mutations or those with family histories suggestive of hereditary cancer. This gene testing is not part of the Oncomine 50-gene panel, which in itself if an investigational tool and not part of standard government funded practice in Australia.

We have added a discussion regarding the APC gene in our revised manuscript, but it’s relevance and utility is outside the scope of our manuscript.
